# Functions and mechanisms of UC-MSC-derived exosomal miR-486-5p in pulmonary fibrosis

**DOI:** 10.3389/fonc.2025.1542008

**Published:** 2025-07-07

**Authors:** Xue-feng Shi, Ya-jun Tuo, Zhen-yun Liao, Jie Duo, Hao Yang

**Affiliations:** ^1^ Department of Pulmonary and Critical Care Medicine, Qinghai Provincial People’s Hospital, Xining, China; ^2^ Department of Experimental Medical Science, Ningbo No.2 Hospital, Ningbo, China; ^3^ Department of Pulmonary Medicine, Taian 88 Hospital, Taian, China

**Keywords:** pulmonary fibrosis, stem cells, exosomes, miR-486-5p, fibroblast differentiation

## Abstract

**Background:**

Currently, nintedanib and pirfenidone are the two primary pharmacological agents used to treat pulmonary fibrosis (PF). However, neither of these medications effectively halts the progression of PF or preserves lung function. As a result, lung transplantation remains the sole viable treatment option for patients in the advanced stages of the disease. Therefore, it is imperative to identify new therapeutic agents that can more effectively address this condition.

**Methods:**

Exosomes were harvested from the supernatants of human umbilical cord-derived mesenchymal stem cells (UC-MSCs) transfected with either control or miR-486-5p-overexpressing lentivirus via ultracentrifugation and subsequently resuspended in minimum essential medium (MEM). The immunophenotypes were analyzed by Western blotting, and their concentration was determined using the Nanoparticle Tracking Analysis device, NanoSight NS300. The influence of exosomal microRNA-486-5p (miR-486-5p) derived from UC-MSCs on apoptosis in MRC-5 cells was assessed using flow cytometry, and cell proliferation was evaluated through the CCK-8 assay. The expression levels of miR-486-5p, fibroblast growth factor 9 (FGF9), and extracellular matrix (ECM)-related genes were quantified through quantitative reverse transcription-polymerase chain reaction (qRT-PCR).

**Results:**

MiR-486-5p inhibits TGF-β 1-induced pulmonary fibroblast differentiation by targeting FGF9. Exogenous exosomes facilitate the transfer of miR-486-5p to MRC-5 cells. The presence of exosomal miR-486-5p reduces the mRNA expression of FGF9, fibronectin (Fn), alpha-smooth muscle actin (α-SMA), vimentin, COL1A1, and COL3A1, and decreases FGF9 and vimentin protein levels. Compared to control exosomes, UC-MSC-derived exosomal miR-486-5p slightly promotes apoptosis in MRC-5 cells (*p* = 0.06) but does not significantly affect cell proliferation (*p* > 0.05).

**Conclusion:**

Exosomal miR-486-5p derived from UC-MSCs shows potential therapeutic efficacy in regulating fibroblast differentiation by targeting FGF9, thereby mitigating the progression of PF.

## Introduction

Pulmonary fibrosis (PF) is a progressive and irreversible lung disease affecting millions of individuals and is associated with a poor prognosis (1). The pathological changes in PF primarily involve the pulmonary interstitium and are characterized by reduced lung diffusion capacity and subsequent tissue hypoxia. PF develops from repeated injury to the alveolar epithelium or endothelium, which activates both the innate and adaptive immune systems in an effort to restore the damaged tissue. Studies have shown that transforming growth factor-beta (TGF-β) levels are elevated in the lungs of patients with pulmonary fibrosis, as well as in animal models of the disease. Treating lung fibroblasts or epithelial cells with TGF-β *in vitro* can replicate some key pathological features observed in idiopathic pulmonary fibrosis (IPF) patients, including increased extracellular matrix (ECM) deposition and abnormal cell proliferation (2). Both animal and cell studies have confirmed that elevated TGF-β levels are closely associated with the onset and progression of pulmonary fibrosis. The cellular changes and molecular mechanisms observed in lung cells treated with TGF-β are closely mirror the pathological processes seen in human pulmonary fibrosis (3). TGF-β induces angiogenesis and activates myofibroblasts, leading to increased production of ECM components such as collagen and fibronectin (4). Failure to regulate the fibrotic response perpetuates the inflammation, resulting in abnormal wound healing, tissue damage, excessive ECM deposition, and ultimately lung scarring (fibrosis). Lung fibrosis impairs oxygen delivery to the blood, leading to decreased lung function and respiratory failure (5).

Mesenchymal stem cells (MSCs) possess the ability to self-renewal and multipotent differentiation into various cell types, including epithelial cells, adipocytes, osteoblasts, and muscle cells. Due to their multipotency, low immunogenicity, and paracrine actions, MSCs have been widely utilized in treating various diseases, including acute and chronic lung injuries (6). Compared to MSCs themselves, MSC-derived exosomes (MSC-Exos) offer significant advantages by effectively minimizing adverse effects. MSC-Exos are emerging as a promising cell-free therapeutic tool, capable of carrying various molecules and protecting their cargo from degradation in the bloodstream (7). Moreover, studies have shown that exosome-carried microRNAs (miRNAs) act as key regulatory elements in intercellular communication (8). Additionally, exosome therapy derived from human bronchial epithelial cells has been reported to inhibit TGF-β-WNT crosstalk in pulmonary fibrosis (9).

In our previous research, we discovered that miR-486-5p plays a role in the hypoxic regulation of lung fibrosis. Hypoxia may influence p-SMAD2 expression by modulating miR-486-5p levels, contributing to the pathogenesis of pulmonary interstitial fibrosis. Consistent with our findings, Ji and colleagues reported that an increase in miR-486-5p expression led to a significant reduction in both the extent and severity of lung lesions, suggesting that miR-486-5p may have antifibrotic properties (10). Given that miR-486-5p expression is reduced in lung fibrotic diseases and may function as an antifibrotic effector in pulmonary fibrosis development, this study aims to investigate the role and underlying mechanisms of the umbilical cord (UC)-MSC-derived exosomal miR-486-5p in pulmonary fibrosis.

## Methods

### Cell lines

Human embryonic lung fibroblast MRC-5 cells were cultured in minimum essential medium (MEM) basal medium supplemented with 10% fetal calf serum, 1% MEM nonessential amino acid, 1% sodium pyruvate, 1% GlutaMAX-1, and 1% penicillin–streptomycin, maintained at 37°C in an incubator with 21% O_2_ and 5% carbon dioxide (CO_2_). Human UC-MSC cells were obtained from Ningbo No. 2 hospital and cultured in a serum-free stem cell culture medium (Lonza, MD, Walkersville, USA).

### Cell transfection

Lentivirus was purchased from GenePharma. MRC-5 and UC-MSC cells were transfected with control lentivirus, miR-486-5p overexpression lentivirus, or miR-486-5p silencing lentivirus, each at a multiplicity of infection (MOI) of 5. Polybrene was added at a concentration of 5 µg/mL to enhance the transfection efficiency. Six hours after transfection, an additional medium was added based on cell condition. Cells were harvested 48 h after transfection for subsequent experiments. Fluorescence microscopy was utilized to assess the transfection efficiency. Moreover, the expression of miR-486-5p was measured by quantitative reverse transcription-polymerase chain reaction (qRT-PCR) to confirm the successful transfection.

### RNA extraction and reverse transcriptase polymerase chain reaction

Total RNA was extracted from MRC-5 cells, UC-MSC cells, or exosomes by using TRIzol reagent (Invitrogen, California, USA) or miRNeasy Mini Kit (Qiagen, Valencia, CA, USA) according to the manufacturer’s instructions. The mRNA levels of fibroblast growth factor 9 (FGF9), Fn, alpha-smooth muscle actin (α-SMA), vimentin, COL1A1, and COL3A mRNA were detected by qRT-PCR using an SYBR Green system and normalized to β-actin. The primers sequence was showed in **Table 1**.

**Table 1:** Primer sequence.

Primer	F	R
FGF9	ACCCAAGAGTGTGTATTCAGAG	AGTGTCCACGTGCTTATATAGG
Fn	AATAGATGCAACGATCAGGACA	GCAGGTTTCCTCGATTATCCTT
α-SMA	CATGAAGTGTGACATCGACATC	TGATCTTGATCTTCATGGTGCT
Vimentin	GTTTCCAAGCCTGACCTCAC	GCTTCAACGGCAAAGTTCTC
N-cadherin	CATCCAGACCGACCCAAACA	AACAGACACGGTTGCAGTTGACT
COL1A1	AAAGATGGACTCAACGGTCTC	CATCGTGAGCCTTCTCTTGAG
COL3A1	TGAAGGGCAGGGAACAACTTGATG	GGATGAAGCAGAGCGAGAAGTAGC

U6 was used as an internal control, and miRNA-486 was measured following the manufacturer’s instructions for the TaqMan MicroRNA Reverse Transcription Kit and TaqMan MicroRNA Assay (Life Technologies, Carlsbad, CA, USA). qRT-PCR analysis was performed using the ABI 7500 Sequence Detector (Applied Biosystems, Foster City, CA, USA).

### Exosome preparation

The UC-MSC cells were transfected with control lentivirus or miR-486-5p overexpression lentivirus. The supernatants were then collected, and large cell debris and membranes were removed by centrifugation. The UC-MSC-derived exosomes were harvested by ultracentrifugation and resuspended in a MEM medium. Their immunophenotypes, including CD9, CD63, CD81, HSP60, HSP90, GRP78, and TSG101, were detected by Western blot analysis. The concentration of exosomes was determined by nanoparticle tracking analysis (NTA) with the NanoSight NS300 (Malvern, UK). The MRC-5 cells were treated with exosomes derived from UC-MSC cells at a concentration of 1 × 10^7^ particles/ml in the medium. The NC exosome group consisted of MRC-5 cells treated with exosomes extracted from UC-MSCs transfected with the control lentivirus, while the miR-486 overexpression exosome group consisted of MRC-5 cells treated with the exosomes extracted from UC-MSCs transfected with the miR-486-5p overexpression lentivirus.

### Western blotting

The MRC-5 cells or stem cell-derived exosomes were lysed in RIPA buffer containing 1% phenylmethanesulfonyl fluoride (PMSF) and 1% phosphatase inhibitors. Protein concentration was measured using the BCA Protein Assay Kit (Thermo Fisher Scientific, Rockford, IL, USA). Twenty micrograms of proteins was loaded onto SDS polyacrylamide gels, transferred to nitrocellulose membranes, and incubated with primary antibodies (anti-CD9, anti-CD63, anti-CD81, anti-HSP60, anti-HSP90, anti-GRP78, and anti-TSG101). Immunoreactive bands were detected with anti-rabbit or anti-mouse peroxidase-conjugated secondary antibodies and visualized by chemiluminescence. The protein levels were analyzed by the ImageJ Software.

### Dual-luciferase reporter assay

The sequence of miR-486-5p and the corresponding FGF9 mutants were cloned and inserted into the pmirGLO vector. In six-well plates, 293 T cells were cultured to approximately 70%–80% confluence and then co-transfected with mutant luciferase or wild-type reporter vector, along with mimic miRNAs or negative control (NC) (2  µg). After 48 h, luciferase activity was measured and normalized.

### Small interfering RNA transfection

Small interfering RNA (siRNA) targeting FGF9 and a nontargeting control were synthesized by GenePharma company and transfected into cells at a final concentration of 50 nM. To prepare the transfection mixture, 125 µl of 50 nM siRNA in Opti-MEM was slowly added to 125 µl Lipofectamine^®^ 3000 (Thermo Fisher Scientific) diluted in Opti-MEM mixture. The mixture was gently pipetted up and down or inverted to mix well, then incubated at room temperature for 15–20 min to allow the siRNA to fully bind with Lipofectamine 3000 and form a complex. Add the incubated siRNA-Lipofectamine 3000 complex drop-by-drop into the cells in a six-well plate. Gently swirl the culture plate to evenly distribute the complex on the cell surface. Then, return the culture plate to the incubator maintained at 37°C with 5% CO_2_ for continued cultivation. Then, cells were incubated for 48 h for the following experiment.

### CCK-8 proliferation assay

MRC-5 cells were seeded into 96-well plates at 5,000 cells per well in a 100-µl medium and treated with NC exosomes or miR-486-5p-overexpressing exosomes. After 24 and 48 h of incubation, 10 µl of Cell Counting Kit-8 (CCK-8) solution was added to each well. The plates were then returned to the incubator for 2 h, and the reaction was measured with a microplate reader.

### Apoptosis assays

A total of 2 × 10^5^ MRC-5 cells/well were seeded in six-well plates to grow overnight. The culture supernatant was then replaced with fresh serum-free media containing either NC exosomes or miR-486-5p-overexpressing exosomes. After treatment, cells and cell culture supernatants were harvested and washed twice with phosphate-buffered saline (PBS). The degree of apoptosis was analyzed using the Annexin V/APC Apoptosis Assay Kit according to the manufacturer’s instructions. Flow cytometry analysis was performed using FACS Calibur (BD Biosciences, Shanghai, China).

### Statistical analysis

All values are presented as mean ± SD. Comparisons between the two groups were analyzed via Student’s *t*-tests. Differences between groups were considered statistically significant at *p* < 0.05.

## Results

### MiR-486-5p blocks TGF-β1-induced pulmonary fibroblast differentiation

**Figure 1:**
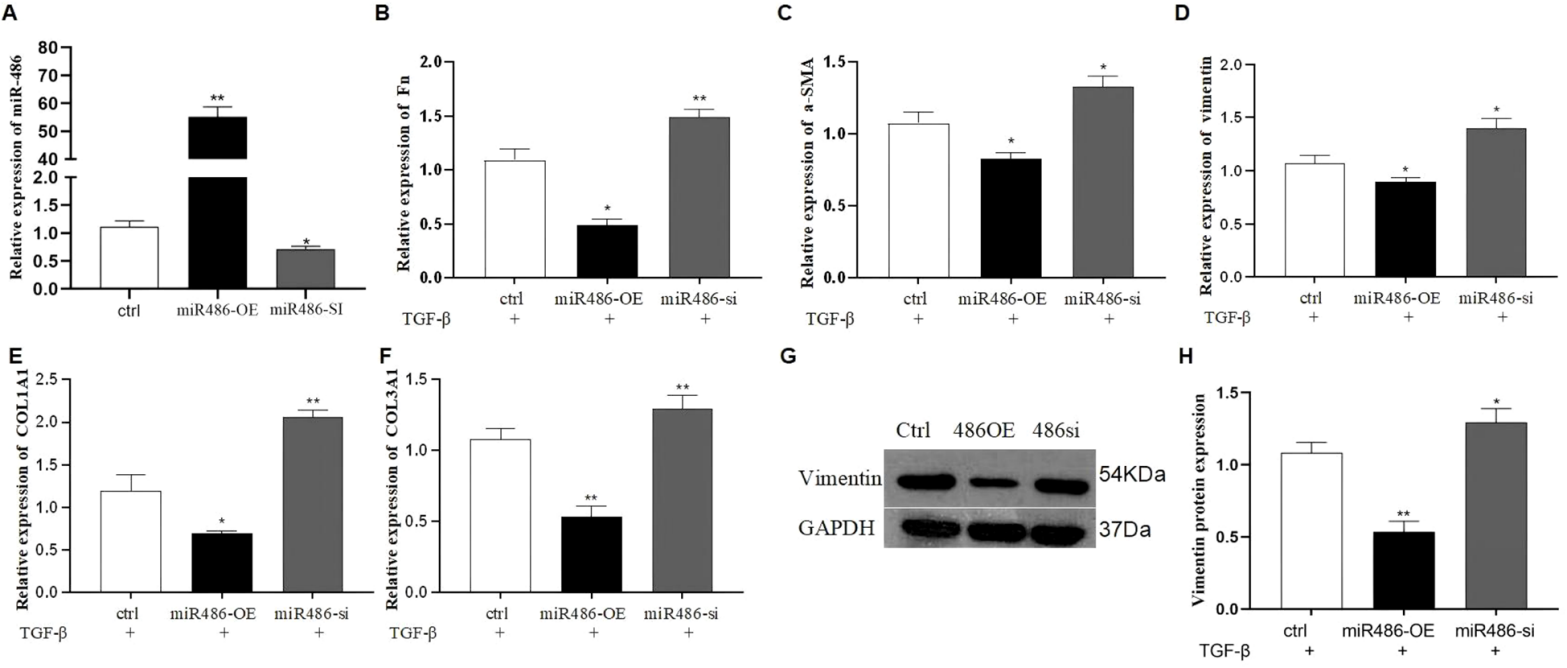
The effect of miR-486-5p on TGF-β1-induced fibroblast differentiation in MRC-5 cells. **(A)** MiR-486-5p mRNA expression in TGF-β1-induced MRC-5 cells transfected with miR-486 overexpression lentivirus, miR-486 downexpression lentivirus, or control lentivirus. **(B)** Fn mRNA expression in TGF-β1-induced MRC-5 cells transfected with miR-486 overexpression lentivirus, miR-486 downexpression lentivirus, or control lentivirus. **(C)** α-SMA mRNA expression in TGF-β1-induced MRC-5 cells transfected with miR-486 overexpression lentivirus, miR-486 downexpression lentivirus, or control lentivirus. **(D)** Vimentin mRNA expression in TGF-β1-induced MRC-5 cells transfected with miR-486 overexpression lentivirus, miR-486 downexpression lentivirus, or control lentivirus. **(E)** COL1A1 mRNA expression in TGF-β1-induced MRC-5 cells transfected with miR-486 overexpression lentivirus, miR-486 downexpression lentivirus, or control lentivirus. **(F)** COL3A1 mRNA expression in TGF-β1-induced MRC-5 cells transfected with miR-486 overexpression lentivirus, miR-486 downexpression lentivirus, or control lentivirus. **(G)** Vimentin protein expression in TGF-β1-induced MRC-5 cells transfected with miR-486 overexpression lentivirus, miR-486 downexpression lentivirus, or control lentivirus. **(H)** Densitometric analysis of vimentin protein expression from Western blot results. Values are presented as mean ± SD, *n* = 3. * P<0.05, ** P<0.01.

TGF-β1 is essential for PF progression by inducing fibroblast differentiation. To evaluate the effect of miR-486-5p on TGF-β1-induced fibroblast differentiation, mesenchymal markers (fibronectin [Fn], vimentin, and α-SMA) and collagen protein (COL1A1 and COL3A1) were detected in MRC-5 cells transfected with miR-486-5p overexpression or downexpression lentivirus. Results showed that miR-486-5p expression was significantly upregulated in MRC-5 cells transfected with the overexpression lentivirus and downregulated by the inhibition lentivirus (**Figure 1A**). Moreover, overexpression of miR-486-5p inhibited the mRNA expression of Fn, α-SMA, vimentin, COL1A1, and COL3A1, as well as vimentin protein expression in MRC-5 cells treated with TGF-β1 (**Figures 1B–H**). In contrast, inhibition of miR-486-5p in TGF-β1-treated MRC-5 cells upregulated the mRNA expression of Fn, α-SMA, vimentin, COL1A1, and COL3A1, along with vimentin protein expression (**Figures 1B–H**).

### MiR-486-5p blocks TGF-β1-induced pulmonary fibroblast differentiation by targeting fibroblast growth factor 9

**Figure 2:**
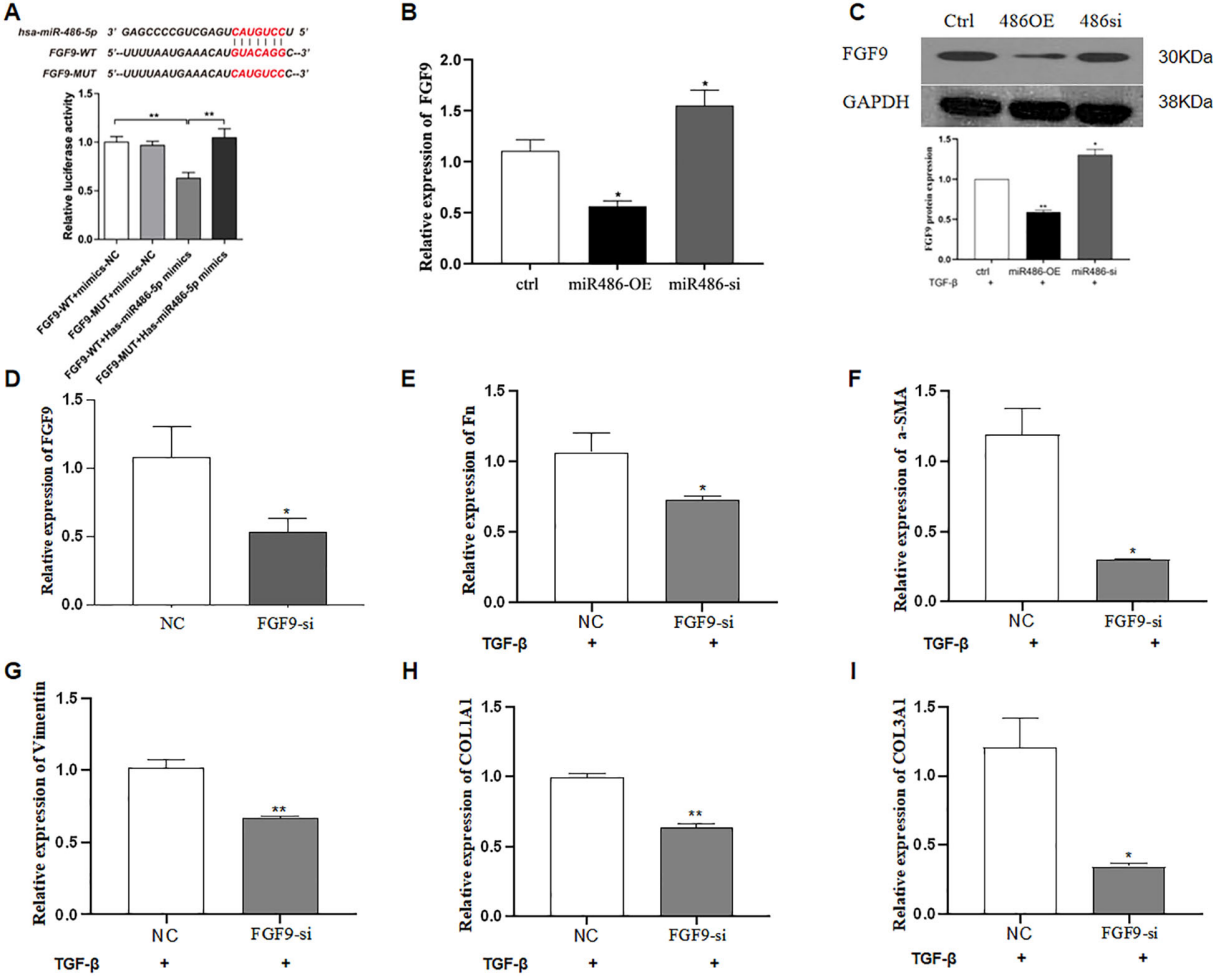
MiR-486-5p blocks TGF-β1-induced fibroblast differentiation by targeting FGF9. **(A)** Validation of FGF9 as a target gene of miR-486-5p using a dual-luciferase reporter assay. **(B)** Validation of FGF9 as a target gene of miR-486-5p (qRT-PCR). **(C)** Validation of FGF9 as a target gene of miR-486-5p by Western blot; the bottom panel shows densitometric analysis of the Western blot results. Values are presented as mean ± SD, *n* = 3. **(D)** FGF9 expression in MRC-5 cells following FGF9 silencing by siRNA transfection. **(E)** Fn expression in MRC-5 cells following FGF9 silencing. **(F)** α-SMA expression in MRC-5 cells following FGF9 silencing. **(G)** Vimentin expression in MRC-5 cells following FGF9 silencing. **(H)** COL1A1 expression in MRC-5 cells following FGF9 silencing. **(I)** COL3A1 expression in MRC-5 cells following FGF9 silencing. * P<0.05, ** P<0.01.

FGF9 was predicted as a target gene of miR-486-5p using TargetScan Release 8.0 (www.targetscan.org). To validate this prediction, a dual-luciferase reporter system was constructed by inserting either the wild type (WT) or mutated (MUT) sequence of FGF9 into the 3′ UTR of the pmirGLO vector (WT). The results showed that co-transfection with the miR-486-5p mimic and WT reporter significantly decreased luciferase activity compared to the NC, confirming the direct interaction between miR-486-5p and the 3′ UTR of FGF9. However, no significant change in luciferase activity was observed after co-transfection with the FGF9 MUT construct and the miR-486-5p mimic (**Figure 2A**). Furthermore, qRT-PCR and Western blot analyses were performed to assess whether miR-486-5p regulates FGF9 expression. The results showed that overexpression of miR-486-5p inhibited both FGF9 mRNA and protein expression (**Figures 2B, C**), whereas, inhibition of miR-486-5p led to upregulation of FGF9 mRNA and protein levels (**Figures 2B, C**). Next, we validated the role of FGF9 in the progression of pulmonary fibrosis. Lipofectamine™ 3000 and siRNA were used to silence FGF9 expression. Silencing FGF9 significantly downregulated its mRNA expression, as well as the expression of Fn, α-SMA, vimentin, COL1A1, and COL3A1 (**Figures 2D–I**).

### Exosomal miR-486-5p regulates fibroblast differentiation by targeting FGF9

**Figure 3:**
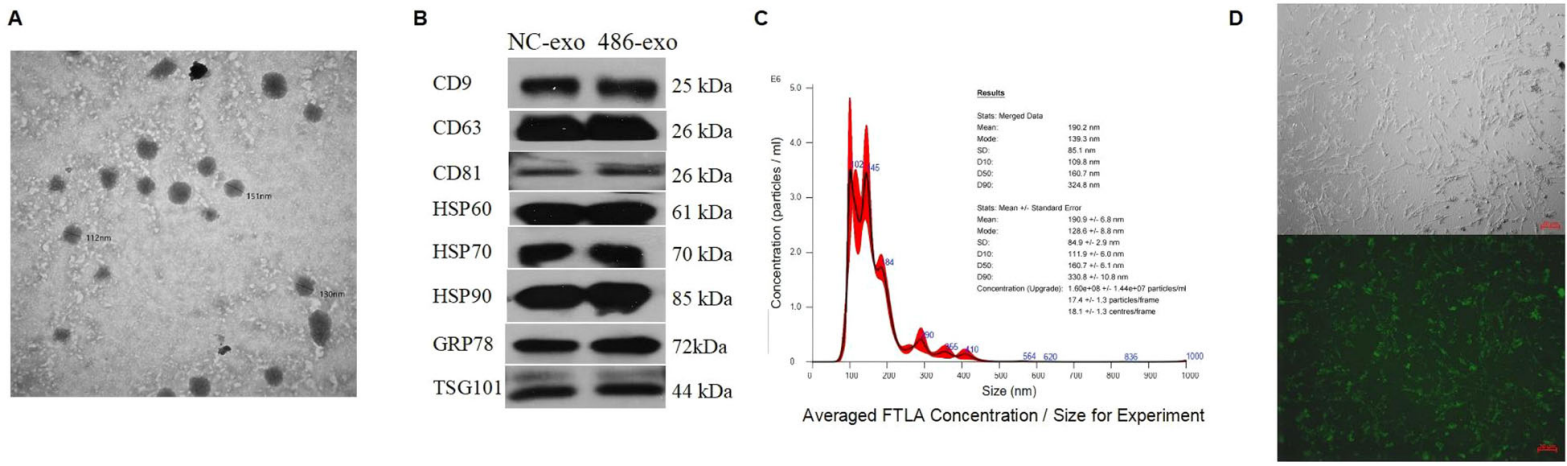
UC-MSC-derived exosome identification and concentration measurement. **(A)** Transmission electron micrographs of exosomes isolated by ultracentrifugation from UC-MSC culture supernatants (diameter < 200 nm). **(B)** Confirmation of exosome presence and purity by Western blot analysis. **(C)** Confirmation of exosome size and concentration by NTA. **(D)** Exosomes clustered around MRC-5 cells (top: optical microscopy; bottom: fluorescence microscopy).

**Figure 4:**
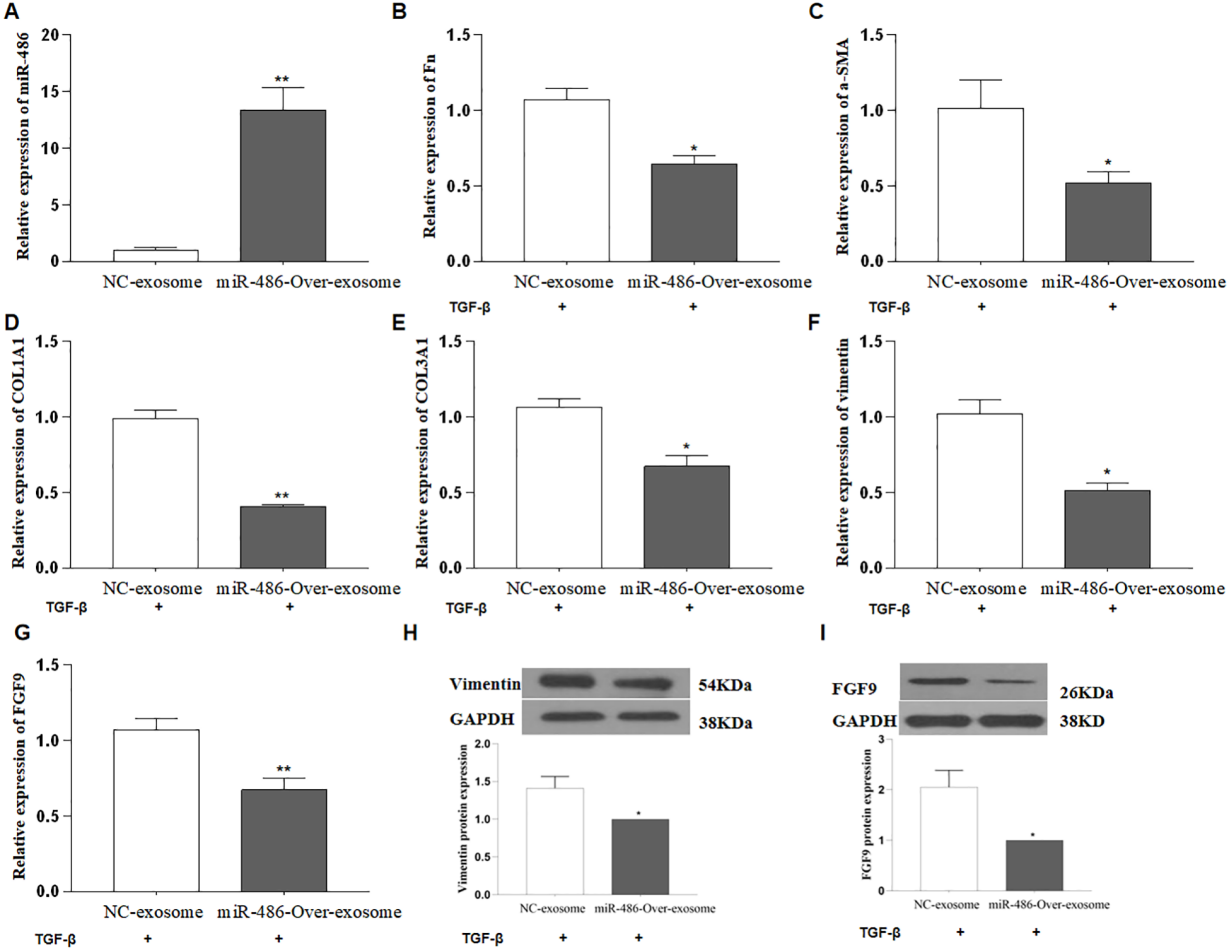
UC-MSC-derived exosomal miR-486-5p blocks TGF-β1-induced fibroblast differentiation by targeting FGF9. **(A)** MiR-486-5p expression in MRC-5 cells treated with UC-MSC-derived exosomal miR-486-5p. **(B)** Fn expression in TGF-β1-induced MRC-5 cells treated with UC-MSC-derived exosomal miR-486-5p. **(C)** α-SMA expression in TGF-β1-induced MRC-5 cells treated with UC-MSC-derived exosomal miR-486-5p. **(D)** COL1A1 expression in TGF-β1-induced MRC-5 cells treated with UC-MSC-derived exosomal miR-486-5p. **(E)** COL3A1 expression in TGF-β1-induced MRC-5 cells treated with UC-MSC-derived exosomal miR-486-5p. **(F)** Vimentin expression in TGF-β1-induced MRC-5 cells treated with UC-MSC-derived exosomal miR-486-5p. **(G)** FGF9 expression in TGF-β1-induced MRC-5 cells treated with UC-MSC-derived exosomal miR-486-5p. **(H)** Vimentin protein expression in TGF-β1-induced MRC-5 cells treated with UC-MSC-derived exosomal miR-486-5p, and the bottom panel shows densitometric analysis of the Western blots. Values are presented as mean ± SD, *n* = 3. **(I)** FGF9 protein expression in TGF-β1-induced MRC-5 cells treated with UC-MSC-derived exosomal miR-486-5p; the bottom panel shows densitometric analysis of the Western blot results. Values are presented as mean ± SD, *n* = 3. * P<0.05, ** P<0.01.

Exosomes are small extracellular vesicles that contain miRNAs, mRNAs, and active proteins, and they play key roles in cellular communication. In this study, human UC-MSCs were transfected with either control or miR-486-5p overexpression lentivirus, after which exosomes were isolated by ultracentrifugation. The size of the exosomes ranged from approximately 30 to 180 nm (**Figure 3A**), and they were characterized by the presence of CD9, CD63, CD81, HSP60, HSP70, HSP90, GRP78, and TSG101 (**Figure 3B**). The concentration of exosomes was approximately 1.6 × 10^8^ ± 1.44 × 10^7^ particles/ml (**Figure 3C**). Exosomes were observed to accumulate around MRC-5 cells following treatment (**Figure 3D**). To confirm the transfer of miRNAs via exosomes and investigate the role of miR-486-5p in fibroblast differentiation in PF, MRC-5 cells were treated with exosomes derived from miR-486-5p-overexpressing UC-MSCs and control UC-MSCs. The results showed that treatment with UC-MSC-derived exosomal miR-486-5p increased intracellular miR-486-5p levels in MRC-5 cells (**Figure 4A**), indicating that exogenous exosomes can mediate the transfer to MRC-5 cells. Moreover, exosomal miR-486-5p inhibited the mRNA expression of FGF9, Fn, α-SMA, vimentin, COL1A1, and COL3A1 (**Figures 4B–G**), as well as the protein expression of FGF9 and vimentin (**Figures 4H, I**).

### Exosomal miR-486-5p on the proliferation and apoptosis of MRC-5 cells

**Figure 5:**
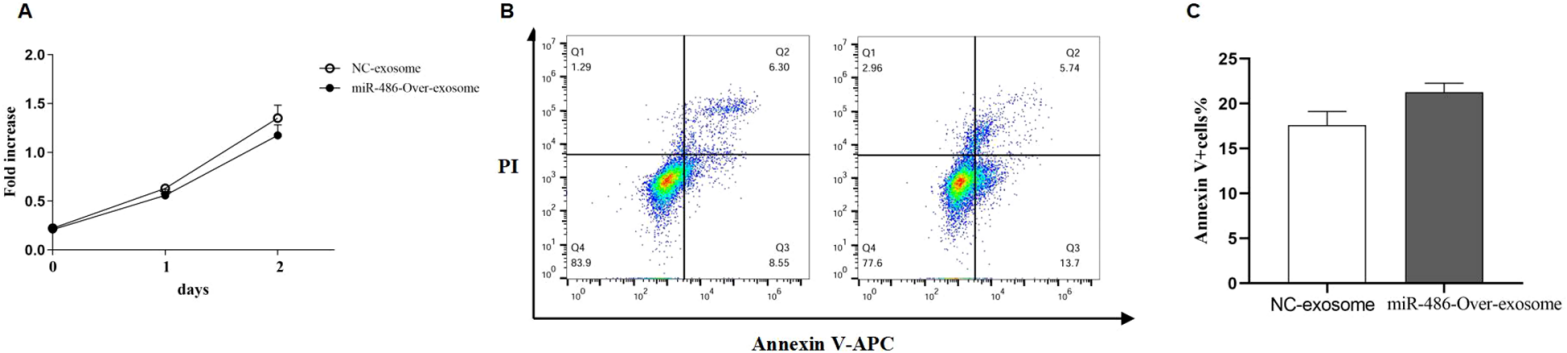
The effect of UC-MSC-derived exosomal miR-486-5p in MRC-5 cell proliferation and apoptosis. **(A)** The effect of UC-MSC-derived exosomal miR-486-5p on MRC-5 cell proliferation. **(B)** The effect of UC-MSC-derived exosomal miR-486-5p on apoptosis of MRC-5 cells. **(C)** The effect of UC-MSC-derived exosomal miR-486-5p on the apoptosis of MRC-5 cells. Values are presented as mean ± SD, *n* = 3.

Excessive proliferation of pulmonary fibroblasts is one of the primary pathological features of pulmonary fibrosis. To evaluate the effect of exosomal miR-486 on the biological functions of MRC-5 cells, cell proliferation, and apoptosis were assessed using the CCK-8 assay and Annexin V staining. As shown in **Figures 5A–C**, compared to control exosomes, UC-MSC-derived exosomal miR-486-5p modestly promoted apoptosis in MRC-5 cells (*p* = 0.06) but had no significant effect on their proliferation (*p* > 0.05).

## Discussion

PF is a fatal interstitial lung disease characterized by progressive difficulty in breathing and impaired lung function. The pathogenesis of PF remains unclear. Currently, the primary pharmacological treatments are nintedanib and pirfenidone; however, neither effectively halts PF progression or preserves lung function. Lung transplantation remains the only effective option for patients with end-stage PF. Therefore, identifying new therapeutic agents for PF patients is imperative.

MiRNAs are small molecules that play crucial roles in the development of various lung diseases, including PF and hold promise as therapeutic targets. One miRNA of particular interest is miR-486-5p, which has been found to be downregulated in the lung tissues of patients with IPF and in the serum of patients with silicosis. Animal studies have also shown that miR-486-5p can inhibit fibrosis. Therefore, miR-486-5p may function as an antifibrotic miRNA and help attenuate the progression of fibrotic lung disease. In this study, we aimed to investigate the role of miR-486-5p in the development of pulmonary fibrosis. We overexpressed miR-486-5p in MRC-5 cells, a cell line commonly used in fibrosis research, and observed a decrease in the expression of fibrosis genes, including Fn, α-SMA, vimentin, COL1A1, and COL3A1. This finding aligns with previous studies that have identified miR-486-5p as an antifibrotic miRNA (10).

Furthermore, we employed bioinformatics tools such as TargetScan to predict potential targets of miR-486-5p. FGF9 emerged as a candidate, with its expression localized to specific populations of myofibroblasts within the fibroblastic foci, as well as in the hyperplastic and hypertrophic alveolar epithelium (11). We confirmed FGF9 as a direct target of miR-486-5p using luciferase reporter assays, Western blotting, and quantitative -time polymerase chain reaction (qRT-PCR). These results indicated that miR-486-5p regulates fibroblast differentiation by targeting FGF9.

Stem cells possess regenerative capabilities and significant potential for multidirectional differentiation. The therapeutic effects of stem cell therapy have been demonstrated in numerous studies. Although cell replacement was initially considered the primary mechanism underlying these effects, it is now understood that the paracrine effects of stem cells play a crucial role in their therapeutic function (12). In this context, extracellular vesicles, particularly exosomes, have emerged as key components of the paracrine mechanism involved in stem cell therapy. Exosomes are nanosized vesicles that carry RNA, miRNAs, lipids, and proteins, and they play important roles in the pathogenesis of lung diseases (13–15). Moreover, they hold therapeutic potential for conditions such as PF, allergic asthma, and lung cancer (16–18). Additionally, stem cell-derived exosomes exhibit lower immunogenicity than their parental stem cells and offer advantages such as ease of storage and the ability to regulate the cellular environment, making them a promising novel potential approach for disease treatment. Given the effects of stem cell-derived exosomal miR-486 on erythroid differentiation and muscle atrophy (19, 20), as well as the potential of MSC-derived exosomes in preventing pulmonary fibrosis, we aim to elucidate the role of UC-MSC-derived exosomal miR-486-5p in the progression of PF (21, 22). Harnessing the therapeutic potential of UC-MSC-derived exosomal miR-486-5p holds great promise for the treatment of pulmonary fibrosis. Our objective is to confirm that exosomal miR-486-5p derived specifically from UC-MSCs exhibits superior therapeutic effects compared to exosomes from UC-MSCs in general. By conducting further research and experiments, we aim to gain a deeper understanding of the mechanisms underlying the therapeutic benefits of exosomal miR-486-5p and contribute to the development of new treatment strategies for pulmonary fibrosis. This research has the potential to make a significant impact in the field of respiratory medicine and improve the quality of life for patients suffering from this debilitating disease.

PF is characterized by abnormal remodeling of lung tissue, involving excessive fibroblast proliferation and ECM deposition (23). Our findings demonstrate that exosomal miR-486-5p derived from UC-MSCs inhibits the expression of fibrosis-related genes, including Fn, α-SMA, vimentin, COL1A1, and COL3A1. This inhibition may ultimately prevent fibroblast differentiation and excessive ECM accumulation. Importantly, the inhibitory effect of UC-MSC-derived exosomal miR-486-5p was superior to that of control UC-MSC-derived exosomes.

In conclusion, our study demonstrates that UC-MSC-derived exosomal miR-486-5p regulates fibroblast differentiation by targeting FGF9, ultimately preventing the progression of pulmonary fibrosis. The therapeutic potential of UC-MSC-derived exosomal miR-486-5p surpasses that of control UC-MSC-derived exosomes. These findings provide valuable insights for developing novel therapeutic strategies to treat pulmonary fibrosis.

## Data Availability

The raw data supporting the conclusions of this article will be made available by the authors, without undue reservation.
